# Bromodomain and Extra-Terminal Protein Inhibitors: Biologic Insights and Therapeutic Potential in Pediatric Brain Tumors

**DOI:** 10.3390/ph15060665

**Published:** 2022-05-26

**Authors:** Andrew Groves, Jessica Clymer, Mariella G. Filbin

**Affiliations:** 1 Department of Pediatric Oncology, Dana-Farber Boston Children’s Cancer and Blood Disorders Center, Boston, MA 02215, USA; jessicar_clymer@dfci.harvard.edu (J.C.); mfilbin@broadinstitute.org (M.G.F.); 2 Broad Institute of Harvard and MIT, Cambridge, MA 02142, USA

**Keywords:** BET inhibitor, epigenetics, pediatric brain tumors, diffuse intrinsic pontine glioma, medulloblastoma, ependymoma, embryonal tumor with multilayer rosettes, atypical teratoid rhabdoid tumor

## Abstract

Pediatric brain tumors have surpassed leukemia as the leading cause of cancer-related death in children. Several landmark studies from the last two decades have shown that many pediatric brain tumors are driven by epigenetic dysregulation within specific developmental contexts. One of the major determinants of epigenetic control is the histone code, which is orchestrated by a number of enzymes categorized as writers, erasers, and readers. Bromodomain and extra-terminal (BET) proteins are reader proteins that bind to acetylated lysines in histone tails and play a crucial role in regulating gene transcription. BET inhibitors have shown efficacy in a wide range of cancers, and a number have progressed to clinical phase testing. Here, we review the evidence for BET inhibitors in pediatric brain tumor experimental models, as well as their translational potential.

## 1. Introduction

Pediatric brain tumors are the most common solid tumor in children, and are the leading cause of cancer-related death [1]. Several aggressive pediatric brain tumors, such as medulloblastoma (MB), high-grade glioma (HGG), ependymoma (EPN) and atypical teratoid/rhabdoid tumors (ATRTs), are defined by epigenetic dysregulation that has been shown to reflect disordered developmental processes that occur in susceptible cell types and along specific spatio-temporal patterns [2,3]. In the context of normal development, epigenetic mechanisms allow for transcriptional control independent of DNA sequence, and are crucial components of cell differentiation and specialization [4,5,6]. The most widely studied mechanisms include DNA methylation and histone modifications, both of which are commonly co-opted in pediatric brain tumor pathogenesis.

Histones are the fundamental building blocks of chromatin and assemble in octamers, around which DNA is wrapped to form the nucleosome [7]. Each histone has an amino acid tail that extends from the nucleosome and is enriched in positively charged residues, such as lysine and arginine, that are subject to post-translational covalent modifications that have dramatic impacts on chromatin accessibility [8,9]. At least 16 of these modifications have been described to date and include lysine acetylation, lysine and arginine methylation, serine and threonine phosphorylation, and lysine ubiquitination or sumoylation [10,11]. Specific enzymes, known as “writers” and “erasers”, are responsible for catalyzing the transfer of these marks, while “reader” proteins are involved in recognition of the marks to recruit other protein complexes involved in transcriptional control. 

Histone lysine acetylation is one of the most well-studied histone post-translational modification (PTM) and decreases the positive charge of histones leading to a relaxed chromatin conformation associated with greater levels of transcription [12,13]. Histone acetylation writers (histone acetyltransferases, or HATs), erasers (histone deacetylases, or HDACs) and readers (bromodomains, BRDs) have been well characterized and each has been implicated in the pathogenesis of a wide range of cancers [14,15,16,17,18,19]. Over 60 bromodomains have been identified in humans, occurring in over 40 proteins (each containing between 1–6 BRDs) [20]. One important class of bromodomains are the bromodomain and extra terminal (BET) family, that is made up of BRD2, BRD3, BRD4, and BRDT [21]. Each BET protein is characterized by two tandem bromodomains (BD1 and BD2) in their N-terminal region and a C-terminal extra-terminal domain [22,23]. In particular, BRD4 plays a key role in RNA polymerase II dependent transcription through the recruitment of the positive transcription elongation factor complex (P-TEFb) and the Mediator complex to promoter regions [24,25]. Through this activity, BRD4 serves as a critical transcriptional coactivator at regions of hyperacetylation (e.g., enhancers or super-enhancers) and mediates expression of key transcription factors, such as c-MYC [26,27]. Since the discovery of small molecule BET inhibitors in 2010, their activity has been demonstrated in a wide range of transcriptionally addicted human cancers. Here we will review the rationale and translational potential for BET inhibitors in pediatric brain cancers.

## 2. BET Protein Structure and Function

**Figure 1:**
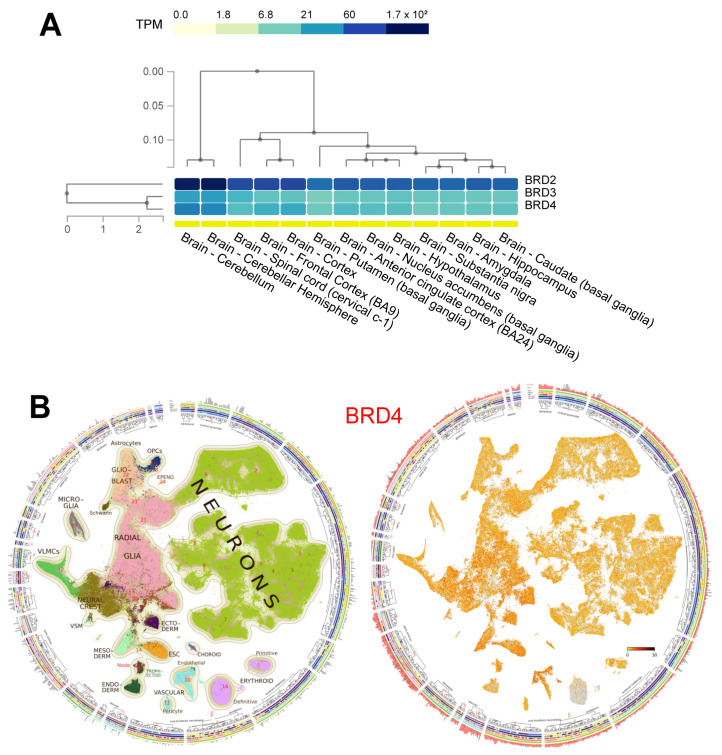
BET protein expression in the central nervous system. (**A**) Human bulk RNA expression from the GTex database, clustered by brain tissue type [34] (**B**) Single-cell RNA sequencing atlas of the developing mouse brain showing ubiquitous BRD4 expression throughout cell types of the developing central nervous system [35,36].

There are four BET proteins, which in humans are referred to as bromodomain-containing protein 2 (BRD2), bromodomain-containing protein 3 (BRD3), bromodomain-containing protein 4 (BRD4), and bromodomain testis associated protein (BRDT). BRD2/3/4 are all ubiquitously expressed, while BRDT is limited to male germ cell tissue. In central nervous system (CNS) tissue, specifically, BRD2/3/4 have all been found to be highly expressed, although expression levels can vary depending on cell-type and spatial location (Figure 1A). BET proteins (particularly BRD2 and BRD4) have also been found to be highly expressed in the developing mouse brain (Figure 1B). Inhibition, via genetic knockout or inhibitor treatment, has been linked with diverse phenotypes, including cerebellar ataxia, seizures, and autism-like behaviors [28,29,30].

BET proteins are comprised of two N-terminal bromodomains (BD1, BD2), an extraterminal domain (ET), and a C-terminal domain (CTD). Each bromodomain contains four alpha helices separated by a variable loop region. This forms a hydrophobic pocket that anchors to acetylated lysine residues via a conserved asparagine residue. The BD1/BD2 amino acid residues critical for acetylated lysine binding are highly conserved across BET proteins; however, there are substantial differences between BD1 and BD2 active sites, which confer functional differences and the ability to chemically target selectively [31]. The BET extra-terminal domain (ET) is able to recruit other chromatin-regulating proteins, such as NSD3, JMJDs, and CHD4 [32]. The C-terminal domain (CTD) is present only in BRD4 and BRDT, and is responsible for recruitment of the positive elongation factor (P-TEFb) [33]. BET proteins utilize these unique structural elements to read acetylated histones at cis-regulatory elements, such as promoters and enhancers, and serve as transcriptional coactivators. Indeed, one of the main mechanisms by which BET inhibitors exhibit anti-cancer effects is through targeting super-enhancer driven oncogenes, such as c-MYC [27].

## 3. BET Inhibitors

Small molecule inhibitors of BET bromodomains were first discovered in 2010 by two groups, based on a thienotriazolodiazepine (JQ1) and benzotriazolodiazepine (I-BET151) scaffold, respectively [37,38]. They were shown to potently inhibit bromodomain binding to acetylated lysine residues, resulting in displacement of BRD4 from nuclear chromatin. Both compounds selectively inhibit BET bromodomains over other bromodomain classes, although neither discriminates between BD1/BD2 within each BET protein nor between BRD2/BRD3/BRD4/BRDT. These compounds have demonstrated impressive pre-clinical efficacy in a variety of cancer models, including BRD4–NUT fusions NUT-midline carcinoma (NMC), acute myeloid leukemia (AML), medulloblastoma, breast cancer, and lung cancer [39,40,41]. In the last decade, several “clinical-grade” pan-BET inhibitors with improved pharmacokinetic profiles have been developed (Table 1). From the initial diazepane-based JQ1 and I-BET151, a variety of scaffolds with potent BET inhibitory properties have been employed successfully. Clinical trials with these agents have shown that thrombocytopenia is an important and common dose-limiting toxicity of pan-BET inhibitors [42].

More recently, BD2 selective BET inhibitors, such as ABBV-744, have been developed with the goal to narrow anti-neoplastic selectivity and reduce off-target hematologic toxicities [43,44]. Another exciting development has been in the field of targeted protein degradation. In 2015, three BET proteolysis-targeting chimeras (PROTACs) were discovered: dBET1, MZ1, and ARV-825 [45,46,47]. Proteolysis-targeting chimeras (PROTACs) are bifunctional small molecules that include a “warhead” ligand that recruits a protein of interest, linked to an E3 ligase ligand, that induces target ubiquitination and subsequent proteasomal degradation. In some settings these compounds have shown enhanced potency in cancer cell lines compared to their parental inhibitors, paving a new strategy for targeting BET proteins in select malignancies.

## 4. BET Inhibitors in Pediatric Brain Tumor Models

### 4.1. Medulloblastoma

Medulloblastoma is the most common embryonal brain tumor occurring in childhood, and accounts for approximately 6.5% of all pediatric brain tumors. Several landmark studies, published in 2006-2011, used genomic profiling to identify distinct molecular subgroups, which were summarized in a 2012 consensus report designating four subgroups (WNT, SHH, Group 3, Group 4) [48,49,50,51,52]. In 2017, three independent groups also used DNA methylation analysis, which recently has been demonstrated to have exquisite discriminatory power in the diagnosis of CNS tumors, to further subclassify medulloblastoma subtypes [53,54,55]. Each medulloblastoma subgroup has unique underlying biology, which is reflected in differences in patient outcomes and treatment responses. These subgroups have subsequently been incorporated into the 2016 and 2021 updated World Health Organization (WHO) Classification of Tumors of the Central Nervous System [56,57].

Treatment for medulloblastoma is aggressive and includes surgical resection, radiation therapy, and chemotherapy. While these treatments have led to five-year overall survival rates over 80%, they can lead to significant treatment-related toxicities, and patients with higher risk features can have survival rates as low as 20% [58]. One subgroup that is associated with a poor prognosis is Group 3 medulloblastoma, which are often metastatic at diagnosis and tend to be refractory to therapy or have late recurrence. Molecularly, Group 3 tumors often have MYC amplification or high MYC expression, and high MYC expression has been reported to be an independent risk factor for poor outcome in medulloblastoma [59].

Several groups have reported promising findings of BET inhibition in MYC-amplified medulloblastoma. The BET inhibitor JQ1 potently decreases cell viability of MYC-amplified medulloblastoma cell lines, causes G1 arrest and apoptosis, and downregulates MYC-targets, as well as MYC transcription itself [60,61]. Furthermore, JQ1 treatment shows in-vivo efficacy with decreased tumor growth and prolonged survival in both flank and cerebellar orthotopic models of Group 3 medulloblastoma (Table 2). JQ1 has also been shown to induce cellular senescence and suppress transcriptional programs associated with poor prognosis in medulloblastoma patients [62].

BET inhibition has also been shown to be a promising strategy in SHH-driven medulloblastoma. Canonical hedgehog (Hh) signaling occurs via binding of Hh ligands to the PTCH1 transmembrane protein, which leads to the cessation of PTCH1′s role in repressing the G protein coupled receptor Smoothened (SMO) [63]. Activation of SMO leads to positive regulation of the GLI zinc-finger transcription factors, leading to the expression of a Hh transcriptional program that regulates proliferation and cell specification [64]. Aberrant Hh signaling has been associated with several cancers, including basal cell carcinoma, medulloblastoma, and pancreatic cancer [65,66]. BRD4 has been shown to be critical in GLI1 and GLI2 transcription via direct promoter occupation using a mouse 3T3 Gli-luciferase reporter cell line [67]. This interaction can be significantly disrupted with JQ1 treatment, providing a biological rationale for BET inhibition in SHH-driven medulloblastoma. Indeed, JQ1 decreases proliferation and viability of SHH-driven medulloblastoma cell lines in-vitro and in-vivo, even when mutations conferring these cell lines to SMO inhibitor resistance are present. The BET inhibitor I-BET151 has also been studied, and was found to significantly attenuate HH activity in the Light2 reporter cell line [68]. Furthermore, I-BET151 decreases growth and viability of a murine derived (Ptch1^+/−^) medulloblastoma cell line in-vitro, decreases GLI1 transcription, and decreases tumor growth in-vivo.

Several synergistic drug combinations with BET inhibitors have also been investigated, with particular focus on cell cycle inhibitors. In 2019, Bandopadhayay et al. used a functional genomic approach including CRISP/Cas9-mediated loss of function and ORF/cDNA rescue screens to identify key regulators of BET inhibitor response and resistance [69]. They found that resistant cells express transcriptional programs associated with neuronal differentiation, while still maintaining proliferative potential. They also demonstrated that CDK4/CDK6 inhibition delays the acquisition of BET inhibitor resistance, and the combination of JQ1 with LEE01 (a CDK4/6 inhibitor) improved survival, compared to monotherapy in flank and orthotopic xenograft models of MYC-driven medulloblastoma. The CDK2 inhibitor Milcilib also synergizes with JQ1 in Group 3 medulloblastoma in-vivo and in-vivo models [70]. From a mechanism standpoint, combination treatment with JQ1 + Milcilib targets MYC expression, as well as MYC stabilization, respectively; the latter being a known effect of CDK2 inhibition through suppression of MYC residue S62 phosphorylation. Through these mechanisms, combination treatment was found to be significantly more effective than either JQ1 or Milcilib monotherapy in downregulating MYC target expression in Group 3 medulloblastoma models.

### 4.2. Diffuse Intrinsic Pontine Glioma

Diffuse intrinsic pontine glioma (DIPG), now referred to as diffuse midline glioma, H3 K27-altered in the 2021 WHO classification, is a lethal brainstem tumor occurring in childhood. Radiation is the only proven therapeutic, despite dozens of chemotherapeutic trials over the last decades, and it only confers a survival advantage of approximately three months [71]. In 2012, a breakthrough was made when several groups reported that over 80% of DIPG tumors harbor a unique lysine-to-methionine mutation (K27M), the H3 histone tail [72,73]. Subsequent work showed that this mutation leads to widespread loss of the repressive H3K27me3 mark through inhibition of the poly-comb repressor 2 complex (PRC2) [74]. Importantly, there are some genomic regions where PRC2 activity is retained and even increased [75].

ChIP sequencing studies have shown that in DIPG, the pathogenic H3K27M mutation colocalizes with acetylated H3K27, RNA polymerase II, and BRD2/BRD4 at sites of active transcription [76]. Treatment with JQ1 impairs the growth and viability of DIPG cell lines, with RNA sequencing demonstrating specific targeted effects at genes with BRD2/BRD4 promoter occupancy. Furthermore, 10 days of JQ1 treatment was found to significantly prolong survival in a DIPG mouse orthotopic (brainstem) patient derived xenograft model. Nagaraja et al. found similar promising findings with JQ1 treatment in a panel of H3K27M mutant DIPG cell lines, while a H3WT glioblastoma cell line (SU-pcGBM2) showed little response [77]. Transcriptomic analysis after JQ1 treatment showed downregulation of genes associated with central nervous system development, such as NTRK3, ASCL1, and MYT1. Lentiviral shRNA knockdown of BRD4 in two DIPG models decreased tumor growth and prolonged survival after mouse orthotopic brainstem injections with lentiviral-modified and control cell lines.

Drug combinations with BET inhibitors have also been investigated in DIPG. Wiese et al. examined JQ1 with the CREB binding protein (CBP) inhibitor ICG-001 [78]. CBP’s main function is as an acetyltransferase which regulates H3K27 acetylation, although it also recruits transcription factors and serves a scaffolding role in multi-unit transcriptional complexes [79]. The authors found that JQ1 + ICG-001 treatment synergistically inhibited DIPG proliferation and invasion potential in DIPG cell lines. RNA sequencing showed that JQ1 downregulated a significant proportion of super-enhancer regulated genes, while surprisingly ICG-001 monotherapy led to an upregulation of these genes. Combination therapy was able to reverse this subset of SE-associated genes which were inadvertently increased with ICG-001 treatment. Another study used H3K27M/PDGFB expressing NSCs as a DIPG model, and showed that JQ1 synergized with the EZH2 inhibitor EPZ6438 (tazemetostat) in-vitro and in-vivo [80]. Taylor et al. showed that targeting the NOTCH pathway with the γ-secretase inhibitor MRK003 also synergized with JQ1 in 2/3 DIPG cell lines tested [81].

Recently, high-throughput chromosome conformation capture (Hi-C) has been used to characterize the 3-dimensional chromatin structure of DIPG cells [82]. This technology enables mapping of tumor-specific regulatory networks, as well as enhancer hijacking events. The BET inhibitor BMS986158 and the BET degrader dBET6 were found to significantly perturb the micro- and macro-chromatin interactions of DIPG cells, and these findings were uniformly more pronounced with dBET6 treatment. This study suggests one possible advantage of targeted protein degradation over catalytic inhibition in this context.

### 4.3. Ependymoma

Ependymoma is a brain tumor that occurs in both children and adults and arises from ependymal cells lining the ventricular system and spinal canal. Ependymoma is the third most common brain tumor in childhood, and can arise anywhere along the craniospinal axis; although in children they tend to occur more commonly in a supratentorial (ST) or posterior fossa (PF) location [83]. Diagnosis of ependymoma is made histologically with three distinct histologic grades (Grade I, II, and III), although prognostic utility is debated given inter-observer variability and the advent of molecular stratification [84]. Standard of care involves maximum safe surgical resection with post-operative radiotherapy [85]. The role of chemotherapy is controversial, although it is commonly used in patients under 18 months of age to delay radiation and there are ongoing efforts to understand if there are benefits to adjuvant and/or maintenance chemotherapy regimens for select patients [86].

In a landmark 2015 study, Patjler et al. used DNA methylation and transcriptomic profiling to identify at least nine clinically and biologically relevant distinct subgroups, which have had major implications in the official WHO classification [87]. Supratentorial ependymomas are made up of ST-sub-ependymoma and two subgroups, defined by characteristic oncogenic fusions (YAP1 and RELA). Of note, RELA ependymomas have been renamed in the most recent WHO classification to ZFTA-fusion positive with the recognition that C11orf95/ZFTA fusions can occur with non-RELA partner genes, such as MAML2/3, NCOA1/2, MN1, or CTNNA2 [88,89,90]. Posterior fossa ependymomas are comprised of PF-sub-ependymoma and two subgroups, denoted group A (PFA, associated with EZHIP overexpression and poor clinical outcome) and group B (PFB, associated with relatively favorable prognosis). Spinal ependymomas are made up of SP-sub-ependymoma, SP myxopapillary ependymoma, and SP ependymoma. A recent study by Bockmayr et al. demonstrated that SP myxopapillary ependymoma is molecularly comprised of two distinct subgroups, MPE-A and MPE-B; with the former being associated with significantly higher relapse rate and decreased progression-free survival [91].

Work by Mack et al. used H3K27ac ChIP-seq to identify enhancers and super-enhancers in a cohort of 42 primary intracranial ependymomas [92]. They found that genetic knockout of super-enhancer regulated genes impaired growth of ependymoma cell lines in-vivo, which provided a rationale for trialing BET inhibition as a therapeutic strategy. They subsequently showed that JQ1 inhibited the growth of two ependymoma cell lines, one PF-A and the other ST-ZFTA. Another group tested the CNS-penetrant BET inhibitor OTX-015 in three ependymoma cell lines (two PFA, one ST (subgroup not specified)) and similarly found it to be effective with sub-micromolar IC50 values in three-day viability assays [93]. They found that in-vivo treatment prolonged survival in 1/2 of the PFA intracranial patient-derived xenograft models tested.

### 4.4. Embryonal Tumor with Multilayer Rosettes (ETMR)

ETMR is a rare, aggressive CNS embryonal tumor occurring primarily in children younger than three years of age [94]. It was first recognized as a distinct molecular diagnosis after studies identified a unique recurrent microRNA amplification of C19MC on chr19q13.42 in a subset of primitive neuroectodermal tumors, including embryonal tumors, with abundant neuropil and true rosettes (ETANTR), ependymoblastoma, medulloepithelioma and supratentorial primitive neuroectodermal tumors (sPNET) [95,96,97]. Treatment of ETMR involves maximum safe surgical resection, and given the young age at diagnosis craniospinal irradiation is typically contraindicated (although local radiation is often used) [98]. High-dose chemotherapy protocols using agents with known embryonal activity, such as cyclophosphamide, vincristine, methotrexate, etoposide, cisplatin, carboplatin, and thiotepa, are often used [99]. However, given the rarity of the disease no standardized treatment protocol has been established yet. Despite these intensive treatment regimens prognosis remains dismal, with reported five-year survival rates between 0–30%.

In 2019, Sin-Chan et al. comprehensively profiled over 80 primary ETMR samples using global methylation, SNP, transcriptional, and miRNA profiling [100]. Through this analysis they uncovered a C19MC-LIN28A-MYCN feed forward oncogenic circuit. LIN28A had previously been found to be highly expressed in ETMR, and is a pluripotency factor and RNA-binding protein important to neural development. LIN28A has also been implicated in the pathogenesis of many advanced human malignancies, including ovarian carcinoma, germ cell tumors, and Wilms’ tumor [101]. The authors hypothesized that this C19MC-LIN28A-MYCN core regulatory circuit could be targeted by BET protein inhibition, and, indeed, found that treatment with JQ1S (the active isomer of JQ1) led to a reduction in viability of ETMR cell lines, and qRT-PCR and western blot showed downregulation of MYCN and LIN28A.

### 4.5. Atypical Teratoid/Rhabdoid Tumor (ATRT)

ATRT is a rare, highly aggressive brain tumor of early childhood [102]. Biallelic inactivation of SMARCB1, a core member of the SWI/SNF (also known as BAF) chromatin remodeling complex, is the core genetic driver event in the vast majority of cases [103,104]. While ATRT has been shown to have a “quiet genome” with few other identifiable pathogenic alterations, this genetic simplicity belies the clinical aggressiveness of these tumors and the difficulties in finding effective targeted therapeutics [105]. Recent studies have used transcriptomics and DNA-methylation to group ATRT into three distinct subgroups: ATRT-TYR, ATRT-SHH, and ATRT-MYC [106,107,108].

ATRT expresses high levels of c-MYC, and ChIP-sequencing shows that SMARCB1 loss leads to significant enrichment of MYC chromatin occupancy at transcriptional start sites (TSS), compared to normal embryonic stem cells [109]. Genetic knockdown of c-MYC decreases ATRT cell growth in-vivo and prolongs survival of mouse PDX models in-vivo, providing a rationale for BET inhibition in this malignancy. Indeed, JQ1 treatment significantly decreases c-MYC transcription, as well as MYC-driven stemness programs regulated by SOX2, Nanog and OCT4 in ATRT. Furthermore, JQ1 treatment significantly prolongs survival in orthotopic xenograft mouse models.

**Table 2:** Findings from pre-clinical studies of BET inhibitors in pediatric brain tumor models.

Tumor	In-Vivo Cell Line/Model	BET Inhibitor	Notable Findings	Citation
MB	MB002, Group 3 MB	JQ1	JQ1 was effective in broad panel of MB cell lines, induced apoptosis and G1 cell cycle arrest. RNA sequencing showed decreased MYC and MC-target expression. Orthotopic xenograft (cerebellar) showed increased survival with JQ1 treatment	Bandopadhayay, [60]
MB	HD-MB3, MYC amplified Group 3 MB	JQ1	JQ1 effective in broad panel of cell lines, inducing apoptosis and G1 cell cycle arrest. Caused decreased MYC and MYC-targets’ expression, and affected components of p53 and cell cycle pathway. Flank xenograft study showed decreased tumor growth and prolonged survival	Hennsen et al. [61]
MB	DAOY, MYC-driven MB	JQ1	JQ1 effective in MB cell lines, induced apoptosis and cell cycle arrest. They also showed that it induced cellular senescence, and that transcriptional programs suppressed by treatment are associated with adverse risk in MB patients. Flank xenograft study showed decreased tumor growth	Venkataraman et al. [62]
MB	MED1-MB, SMO-WT/SMO-D477G-MB (autochthonous derived from *Ptch*^+/−^; *Tpr53*^−/−^ and *Ptch*^+/−^; *lacZ* mice, respectively	JQ1	JQ1 decreased proliferation and viability of SHH-driven MB in-vitro and in-vivo (flank and cerebellar models used), even when cell lines had SMO inhibitor resistance mutations	Tang et al. [67]
MB	Murine *Ptch*^+/−^ MB model	I-BET151	I-BET151 decreased SHH-driven MB growth in-vivo, and decreased Gli1 expression. I-BET151 was effective in decreasing tumor growth in-vivo (subcutaneous)	Long et al. [68]
MB	D458 and MB002, MYC driven MB	JQ1 + LEE01	CDK4/CDK6 inhibition delayed development of BET inhibitor resistance. Combination of JQ1 with LEE01 (a CDK4/6 inhibitor) improved survival in flank and orthotopic xenograft models of MYC-driven medulloblastoma	Bandopadhayay, [69]
MB	GTML2 (murine derived Group 3 MB) and MB002	JQ1 + Milcilib	JQ1+ CDK2 inhibitor synergized to induce apoptosis and cell cycle arrest. Combination treatment in-vivo extended survival in two orthoptic (cerebellar) models of Group 3 MB	Bolin et al. [70]
DIPG	SF8628	JQ1	JQ1 impaired DIPG growth and viability in-vivo, and improved survival in an orthotopic mouse PDX model (brainstem)	Piunti et al. [76]
DIPG	SU-DIPG-VI and SF7761	BRD4 shRNA	JQ1 decreased growth of DIPG cell lines and downregulated genes associated with CNS development. Lentiviral shRNA knockdown of BRD4 extended survival of mice bearing two different orthotopic (brainstem) DIPG models	Nagaraja et al. [77]
DIPG	N/A (no in-vivo data)	JQ1 + ICG-001	BET + CBP inhibition synergized to decrease growth and viability of DIPG cell lines, and preferentially downregulated super-enhancer genes	Wiese et al. [78]
DIPG	H3K27M/PDGFB expressing NSCs	JQ1 + Tazemtostat	BET + EZH2 inhibition was a synergistic combination in H3K27M/PDGFB transformed NSCs	Zhang et al. [80]
DIPG	N/A (no in-vivo data)	JQ1 + MRK003	BET inhibition + NOTCH inhibition synergized in 2/3 DIPG models to induce apoptosis and cell death	Taylor et al. [81]
DIPG	N/A (no in-vivo data)	BMS986158, dBET6	BET inhibition and degradation significantly altered the chromatin architecture of DIPG via Hi-C analysis, although the effect was more pronounced with BET degradation	Wang et al. [82]
Ependymoma	N/A (no in-vivo data)	JQ1	JQ1 inhibited proliferation and viability of one supratentorial (H.EP1) and one PF-A (H.612) ependymoma cell line	Mack et al. [92]
Ependymoma	EPP-MI and EPV-FL-MI (PFA)	OTX015	OTX015 induced apoptosis and cell cycle arrest in two PFA and one ST (subtype not specified) models of ependymoma. In-vivo OTX015 extended survival of the EPP-MI orthotopic intracranial PDX model, but had no improvement in the EPP-FL-MI model	Servidei et al. [93]
ETMR	N/A (no in-vivo data)	JQ1S (active isomer of JQ1)	JQ1S decreased growth and viability of ETMR cell lines in-vivo, and downregulated MYCN and LIN28A expression	Sin-Chan et al. [100]
ATRT	MAF-737	JQ1	JQ1 potently inhibited viability of ATRT (MYC subtype) cell lines, and decreased transcription of c-MYC targets and c-MYC itself. JQ1 prolonged survival in an orthotopic (cerebellar) ATRT model	Allimova et al. [109]

ATRT = atypical teratoid rhabdoid tumor, DIPG = diffuse intrinsic pontine glioma, ETMR = embryonal tumor with multilayer rosettes, MB = medulloblastoma.

## 5. BET Inhibitors in the Clinic

### 5.1. BET Inhibitors in CNS Malignancies

Despite the development of several clinical grade BET inhibitors, few have demonstratable CNS penetrance. While JQ1 is brain penetrant and has widely been used in the pre-clinical setting, it’s poor pharmacokinetic properties (namely short half-life) have precluded translation. One CNS penetrant BET inhibitor that has been well characterized is OTX015/MK-8628/Birabresib, which was shown to be effective in glioblastoma (GBM) pre-clinical models [110]. This led to a Phase IIa trial in patients with recurrent glioblastoma (NCT02296476). Twelve patients were enrolled, and the drug was well tolerated with pharmacokinetic studies demonstrating biologically active levels; however, all patients progressed with a median progression-free survival of two months and the trial was terminated [111]. BMS-986378/CC-90010 is an orally bioavailable, CNS-penetrant BET inhibitor that is currently being evaluated in clinical trials. A phase I trial of CC-90010 in patients with advanced solid tumors and relapsed/refractory non-Hodgkin’s lymphoma enrolled a total of 69 patients, of whom 10 had high-grade gliomas (NCT03220347). There was an overall response rate of 2.9% (*n* = 2), with 8.8% (*n* = 6) of patients achieving stable disease [112]. One patient with a progressive grade II diffuse astrocytoma had a complete response (CR). There are two ongoing studies of CC-90010 in high-grade glioma, including a phase I trial to evaluate the CNS penetration of CC-90010 in patients with progressive/recurrent astrocytoma, anaplastic astrocytoma or GBM (NCT04047303), as well as a Phase Ib study of CC-90010 in combination with temozolomide and radiation in patients with newly diagnosed GBM (NCT04324840).

### 5.2. BET Inhibitors in Pediatrics

In 2019, the first pediatric trial of BET inhibition opened (NCT03936465) investigating the inhibitors BMS-986158 (Arm 1) and BMS-986378/CC-90010 (Arm 2). The primary aims of the trial are to determine toxicities and recommended phase II doses for these agents, while secondary outcomes include: (a) efficacy, (b) pharmacokinetics, and (c) a host of pharmacodynamic and predictive biomarkers. Patients must be <21 and able to swallow intact pills. Each arm has two cohorts, one being with unselected biology (relapsed/refractory solid tumors or lymphoma for Arm 1, CNS tumors for arm 2) and the other enriched for predictive biology. Eligibility for this second criterion includes: MYCN amplification or high copy number gain, MYC amplification or high copy number gain, translocation involving MYC or MYCN, translocation involving BRD4 or BRD3, BRD4 amplification or high copy number gain, and/or histologic diagnosis of NUT midline carcinoma (NMC).

The state of BET inhibitor development in pediatric oncology was recently summarized at a strategy forum organized by ACCELERATE, in collaboration with the European Medicines Agency (EMA) and the US Food and Drug Administration (FDA) [113]. Given that most adult trials of pan-BET inhibitors have been challenged by similar issues (narrow therapeutic index, due to thrombocytopenia, modest anti-tumor activity as monotherapy), it was agreed that opening additional trials to study pan-BET inhibitors, other than BMS-986158 and BMS-986378/CC-90010, was not a worthwhile strategy. Agents with unique properties, such as BDII-selectivity, dual targets e.g., BET/p300, or improved CNS penetrance were highlighted as areas of particular interest worthy of further investigation.

## 6. Conclusions

The discovery and development of BET inhibitors was a landmark finding in cancer epigenetics, and many drugs in this class have advanced to clinical trial stages. Results to date have mostly been mixed, with modest anti-tumor activity and relatively narrow therapeutic index, due to hematologic dose-limiting toxicities. However, there is strong biological and pre-clinical rationale for testing BET inhibitors in pediatric brain tumors, given the common thread of epigenetic dysregulation. The ongoing trial of BMS-986378/CC-90010 in relapsed/refractory pediatric CNS tumors will be highly informative to assess the promise of BET inhibitors in this patient population. Based on experience from experimental models and adult clinical trials, it is likely that monotherapy alone will not be sufficient and, therefore, continued research dedicated to investigating synergistic combinations with BET inhibitors will be critical. 

## Figures and Tables

**Table 1:** BET inhibitors in clinical development.

Agent	Selectivity	Phase	Disease Focus; Comments	Structure
ABBV-075	Pan-BET	I	Solid tumors, AML, multiple myeloma, myelofibrosis	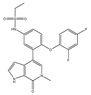
ABBV-744	BDII selective	I	AML, myelofibrosis	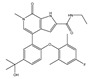
AZDZ5153	Pan-BET	I/II	AML; Unique bivalent binding mode	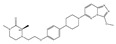
BI-894999	Pan-BET	I	Advanced solid tumors, DLBCL, or NMC	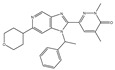
BMS-986158	Pan-BET	I/II	Advanced solid tumors and hematologic malignancies; Ongoing pediatric study	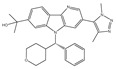
BMS-986378 (CC-90010)	Pan-BET	I	Advanced solid tumors, NHL; Ongoing pediatric study, good CNS penetration	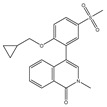
CPI-0610	Pan-BET	I/II	Lymphoma, Multiple myeloma, AML, MPNST	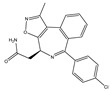
I-BET762 (GSK525762)	Pan-BET	I/II	Hematologic malignancies, solid tumors	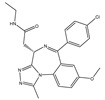
INCB57643	Pan-BET	I/II	Hematologic malignancies, solid tumors	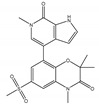
NEO2734	Pan-BET and P300	I/II	Hematologic malignancies, solid tumors	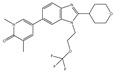
OTX015	Pan-BET	I/II	Hematologic malignancies, solid tumors, GBM	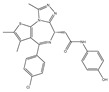
PLX51107	Pan-BET	I	Hematologic malignancies, solid tumors	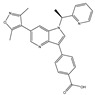

Source: Clinicaltrials.gov (accessed on 10 March 2022); AML = acute myeloid leukemia, DLBCL = diffuse large B-cell lymphoma, GBM = glioblastoma multiforme, NMC = NUT midline carcinoma, MPNST = Malignant peripheral nerve sheath tumor.

## Data Availability

The data presented in this study (Figure 1) is openly available at the Gtex portal [34] and the Mouse Brain Atlas [35,36].
